# In silico approach to designing rational metagenomic libraries for functional studies

**DOI:** 10.1186/s12859-017-1668-y

**Published:** 2017-05-22

**Authors:** Anna Kusnezowa, Lars I. Leichert

**Affiliations:** Institute of Biochemistry and Pathobiochemistry – Microbial Biochemistry, Ruhr University Bochum, Universitätsstr. 150, 44780 Bochum, Germany

**Keywords:** Functional metagenomics, Global ocean sampling project, GOS, Lipase, Protein function

## Abstract

**Background:**

With the development of Next Generation Sequencing technologies, the number of predicted proteins from entire (meta-) genomes has risen exponentially. While for some of these sequences protein functions can be inferred from homology, an experimental characterization is still a requirement for the determination of protein function. However, functional characterization of proteins cannot keep pace with our capabilities to generate more and more sequence data.

**Results:**

Here, we present an approach to reduce the number of proteins from entire (meta-) genomes to a reasonably small number for further experimental characterization without loss of important information. About 6.1 million predicted proteins from the Global Ocean Sampling Expedition Metagenome project were distributed into classes based either on homology to existing hidden markov models (HMMs) of known families, or de novo by assessment of pairwise similarity. 5.1 million of these proteins could be classified in this way, yielding 18,437 families. For 4,129 protein families, which did not match existing HMMs from databases, we could create novel HMMs. For each family, we then selected a representative protein, which showed the closest homology to all other proteins in this family. We then selected representatives of four families based on their homology to known and well-characterized lipases. From these four synthesized genes, we could obtain the novel esterase/lipase GOS54, validating our approach.

**Conclusions:**

Using an in silico approach, we were able improve the success rate of functional screening and make entire (meta-) genomes amenable for biochemical characterization.

**Electronic supplementary material:**

The online version of this article (doi:10.1186/s12859-017-1668-y) contains supplementary material, which is available to authorized users.

## Background

Modern sequencing technology allows for fast and relatively inexpensive sequencing of large amounts of DNA. Whole genome sequencing of genomes of single microorganisms or even whole microbial communities are now state-of-the-art and commercially available. Especially the sequencing of microbial communities, in which the isolation of a single organism is no longer necessary, significantly expanded the number of known proteins in public databases.

The global ocean sampling project (GOS), still one of the largest metagenomic projects to date, was initiated by the J. Craig Venter Institute in 2007 [1]. When the ~6.1 million protein sequences from the GOS dataset were published, it more than doubled the number of known proteins in public databases at that time [2]. Nonetheless, already before the first metagenomic datasets were published, the functional analysis of proteins could not keep up with the speed, with which new gene sequences were discovered. Thus, the functions of most proteins have been, and still are, predicted based on their homology to a much smaller number of well-characterized proteins. Therefore, the function of a major part of all proteins in large data repositories, such as NCBI or EMBL is still unknown (e.g. more than 75% of sequences in Trembl [3]). In some cases, depending on the taxonomic origin, up to 80% of the gene functions of a given organism cannot be inferred from homology [4, 5]. The publication of large metagenomic datasets only exacerbated this challenge.

Within the vast amount of proteins of unknown or predicted function lies great biotechnological potential. Biocatalysts that could be found among those proteins might help us to move away from a petrol-based economy to a more bio-based economy. However, given the sheer size of our databases, it is challenging to make this in silico knowledge of genetic sequences amenable for functional testing in the lab.

Here we present our approach to tackling this challenge using the GOS metagenomic dataset as an example. We leveraged the available information from public databases to classify all proteins of the GOS dataset with known domains into existing families. We classified the remaining set of proteins de novo and devised an algorithm that selects a representative protein sequence from these families based on a minimal phylogenetic distance (i.e. the closest possible relationship) to all other members. Representatives from 4 families containing predicted lipolytic enzymes were functionally characterized in the lab. One protein, termed GOS54, contains the alpha/beta hydrolase domain PF07859 and showed high lipase/esterase activity when expressed in *Escherichia coli*. We demonstrate that our approach can be used to substantially reduce the number of genes from metagenomic datasets that need to be screened for functions. It thus might accelerate biocatalyst discovery.

## Results

### The majority of GOS proteins can be classified based on HMM domains from public protein family databases

The GOS metagenomic project is, to date, still one of the largest publicly available metagenomic datasets. The goal of our study was to make the vast protein sequence diversity contained in this dataset amenable to protein-biochemical functional studies. In a first step, we, therefore, annotated all predicted protein sequences contained in the GOS dataset based on existing HMMs from public protein family databases. We chose two HMM databases: PFAM, a comprehensive protein family database [6] and the complementary, more bacteria-focused TIGRFAMs [7]. HMM searches are fast and can find more distantly related protein family members when compared to standard homology searches such as BLAST [8]. Using an E-value cutoff of ≤ 10^−5^, we could classify, based on the combined PFAM and TIGRFAMs HMMs, 4,436,387 of GOS’s 6,123,395 protein sequences. The use of a less strict cutoff of ≤ 10^−3^ resulted in the additional annotation of less than 4% of proteins in the dataset and an increase in the theoretical number of false positive matches by 2 orders of magnitude. We, therefore, decided to use the more stringent significance threshold for our analysis.

**Fig. 1:**
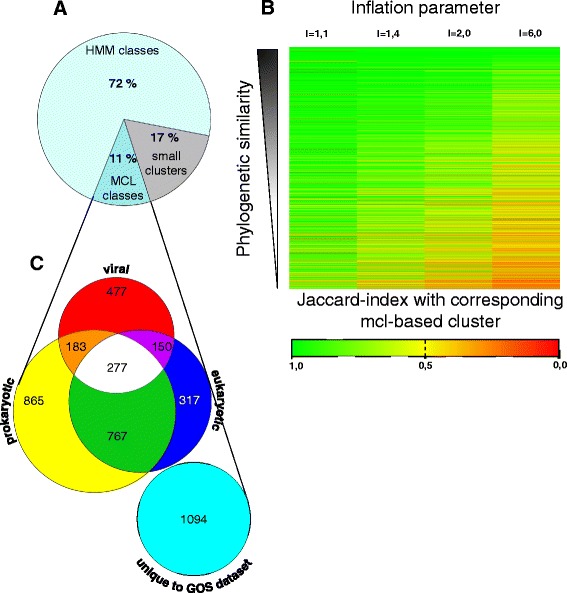
Classification of GOS proteins into families based on existing HMMs from PFAM and TIGRFAMs and de novo MCL clustering. **a** 72% (4,436,387) of the protein sequences in the GOS dataset could be distributed into families based on existing HMMs obtained from PFAM and TIGRFAMs. The remaining 28% (1,687,008) of protein sequences were distributed into MCL-based classes. Of these classes, 680,484 (11% of the total GOS protein sequences) were considered *bona fide* families based on class size, diversity and amount of complete sequences contained therein. **b** To generate MCL-based de novo clusters similar to the clusters based on existing HMMs, the sequences from 2,356 randomly chosen HMM-based families were subjected to Markov Clustering at the indicated inflation parameter values. The HMM-based families were then compared to the resulting MCL-based clusters and the Jaccard similarity coefficient (Jaccard index) was calculated. The MCL-cluster with the highest Jaccard similarity coefficient was considered the cluster corresponding to the HMM-based family. A heatmap was created, with values of the Jaccard indices color-coded according to the legend. The heatmap is sorted by phylogenetic diversity of the HMM-based families. At an inflation parameter of 1.1 the MCL-based clusters showed the highest similarity to the HMM-based families. **c** Taxonomic distribution of MCL-based families. HMMs generated from these families were compared to the RefSeq database and the taxonomic origin of the matching proteins was classified as either of viral, prokaryotic, or eukaryotic origin. More than 1,000 families are specific for the GOS dataset

For the purpose of experimental testability, we ultimately wanted to associate each protein to one, and only one class. However, many proteins matched to multiple HMMs with an E-value that passed our significance threshold. Such an overlap may well be significant, if it was either due to a similarity in the HMMs, or due to a frequent co-occurrence of two distinct domains in proteins of the GOS dataset. If either of those two cases were true, we predicted that a majority of proteins that scored above the threshold for a particular HMM should also score above the threshold for one or more of the other HMMs. We, therefore, considered all proteins that were matching a certain HMM above the threshold as a distinct set. As a measure of the co-occurrence of certain HMMs, we then calculated the Jaccard index for each of these HMM-based sets with all other sets. If the Jaccard index of two sets was above 0.75 (i.e. more than 75% of its combined members were present in both sets), we created a new classifier, which combined both HMMs. In this way, we created classifiers, which were either based on one HMM (13,417 classifiers) or on multiple HMMs (890 classifiers, for the distribution of the number of HMMs in these classifiers, see Additional file 1). We then took each individual protein and considered only the most significant HMM match (i.e. the match with the lowest E-value) and assigned it to the classifier that contained this particular HMM. In this way, we could assign 72% of the proteins in the GOS set to 14,307 HMM-based classifiers, which we then considered protein families (Fig. 1a, Additional files 2 and 3).

### Almost 40% of GOS proteins that do not contain domains recognized by known protein family HMMs were classified de novo

However, 28% of GOS proteins did not match HMMs from PFAM and TIGRFAMs with a significant e-value ≤ 10^−5^ (Fig. 1a). Thus, we clustered these remaining proteins using the Markov Cluster algorithm (MCL) [9]. To obtain MCL-based classes comparable to the classes we annotated using known HMM domains, we adjusted the so-called inflation parameter of this software, which basically adjusts the diversity of the clusters created. Based on a test clustering of ~15% of our HMM annotated proteins we found that an inflation parameter of 1.1 resulted in the largest overlap of MCL-based classes with the existing HMM-based classes and thus this value was used in our subsequent analysis (Fig. 1b).

We then used the MCL algorithm on all proteins from the GOS dataset that could not be annotated using HMMs of known domains. Thus, we distributed these proteins into 148,013 classes containing between 1 and 17,282 sequences (Additional file 4). Theoretically, non-coding DNA can randomly result in open reading frames of significant size. To rule out that we included protein-sequences derived from these “random” open reading frames in our de novo classes, we only considered classes with more than 30 members and no less than 10% complete protein sequences (i.e. sequences derived from DNA sequences with an unambiguous start and stop codon) for further analysis. We argued that the presence of 30 homologous open reading frames excludes that these sequences are random, non-coding DNA. Within this set, we then tested the phylogenetic significance based on multiple sequence alignments. These alignments were generated with MAFFT using default parameters [10] and optimized with MaxAlign [11]. Classes with more than 80% gaps were then excluded, since such large numbers of gaps typically only occur in alignments of proteins with very low to no similarity [11, 12]. For the remaining classes, which we considered *bona fide* families (Additional file 5), we generated Hidden Markov Models. For this purpose, seed alignments were created using MAFFT’s *G*-*INI* strategy with gap regions removed by Gblocks [13]. Low-quality alignments that could not be improved by Gblocks running with relaxed parameters (similar to the parameters described in [14]) were rejected. Amino acids were considered “conserved” if they were present in at least 50% of the sequences at any given position and gaps were only considered if present in more than 50% of the sequences. Multiple sequence alignments fulfilling these criteria were modified by Gblocks as described and were then used as input for HMMER 3.0 to create HMMs [8] (Additional file 6). We then tested the specificity and sensitivity of these HMMs with a test set containing the sequences used to build the HMM (as true positives), and with 80,506 mammalian and plant proteins, matched in size distribution to the (exclusively microbial) GOS protein set (Additional file 7). We then obtained the number of false negatives (i.e. all unmatched proteins from the set of proteins used to build the HMM) and estimated the upper limit of false positives (i.e. all matched mammalian and plant proteins) to calculate the F_1_ score, a measurement of HMM performance [15].

This resulted in a library of 4,130 HMMs with an F_1_ score ≥ 0.5, which contain the information of 680,484 proteins in total (Additional file 8). To test if the high F_1_ scores are the result of overfitting of the data, we tested our approach with a training set derived from the largest of our de novo classes. To this end, we randomly selected two thirds of the sequences contained in FUMEFAM002132, which contains 1177 sequences in total and built an HMM from this subset. We then tested the selectivity and specificity of this HMM against the complete set of proteins in FUMEFAM002132. Repeating this approach 10 times, we consistently achieved an F_1_ score of 1.

We then used this set of HMMs and searched for potential matches in the NCBI RefSeq database, a set of curated prokaryotic, viral, and eukaryotic sequence datasets [16, 17]. By this approach, we found that 477 and 865 of our HMMs were specific for viral or prokaryotic organisms, respectively. 1,094 HMMs did not produce any match in any of these datasets, suggesting that they are specific for the ocean metagenome (Fig. 1c, Additional file 8).

### For each family, we determined one representative sequence

**Fig. 2:**
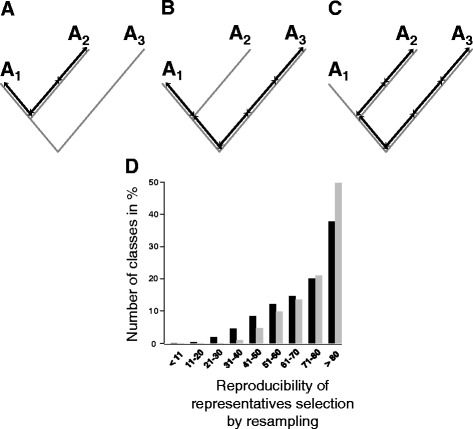
Definition of a representative. **a**-**c**) Schematic overview. The representative of a family is calculated based on the distance in a phylogenetic tree. **a** The phylogenetic distance between sequences A_1_ and A_2_ is 3 units. **b** A_1_ and A_3_ are separated by 4 units. **c** Since the distance between A_2_ and A_3_ amounts to 5 units, the sum of the distances for the three proteins to all other proteins are A_1_: 3 + 4 = 7 units, A_2_: 3 + 5 = 8 units, and A_3_: 4 + 5 = 9 units. Because A_1_ has the shortest distance to all other proteins in the family, it is considered the representative protein. **d** To account for differences in the automatically generated phylogenetic tree, randomly selected subsets containing 90% of the sequences of a family were resampled 100 times. The protein that was selected in these subsets most often as the representative was defined as the representative of the family. The majority of representatives were selected more than 80 times. *Black* bars represent HMM-based families, grey bars MCL-based families

With 14,307 families based on HMMs from public databases and 4,130 novel families, we had the GOS proteins subdivided into 18,437 families in total. To be able to test these families for their biochemical function, we decided to define one protein from each family, which best represents this family. We selected this representative based on its similarity to all other proteins within its family. For this purpose, we first generated a guide tree using MAFFT [10]. We then calculated the sum of the distances in this tree of each individual member to all other members of the class. We reasoned that the member with the minimal sum of distances is the most closely related member to all other members and, therefore, best represents its class (see Fig. 2a-c for a schematic overview of this procedure). Because the representative should be testable in the lab, we considered only complete sequences (i.e. sequences derived from DNA sequences with an unambiguous start and stop codon). To remove bias introduced in the tree building, we randomly created subsets containing 90% of all members and recalculated the guide tree and the associated distances for these subsets 100 times. The member selected most often was then defined as the representative for this family. In this way, we could define one representative for all families containing at least one complete protein, giving us a total of 9,771 representatives (Fig. 2d, Additional file 9).

### Representatives of lipolytic GOS-proteins were tested for activity in the lab

To test the validity of those representatives, we decided to test the representatives of families matching HMMs of well-characterized lipolytic protein families. Lipolytic enzymes such as lipases and esterases constitute an important group of biocatalysts for biotechnological applications [18]. We therefore identified carboxylic ester hydrolases family (EC 3.1.1.-) proteins from the Uniprot database with the highest possible annotation score of 5. After excluding enzymes from potentially pathogenic organisms, we singled out 7 individual proteins, which we then could match, based on homology to four of our families (Table 1).

**Table 1:** Representatives of families matching HMMs of well-characterized lipolytic proteins

Family/Representative	Members with complete sequence	Associated HMM from PFAM	Well-characterized enzymes from UniProt with accession number
FUMEFAM011958/**GOS54**	383	Alpha/beta hydrolase fold PF07859	Acetyl esterase EcE *Escherichia coli* P23872
FUMEFAM010194/**GOS55**	376	GDSL-like Lipase/Acylhydrolase family PF13472	Arylesterase *Streptomyces coelicolor* Q9S2A5, Lipase *Streptomyces rimosus* Q93MW7
FUMEFAM018084/**GOS88**	22	Alpha/beta hydrolase fold PF00561	Pimeloyl-[acyl-carrier protein] methyl ester esterase P13001
FUMEFAM012527/**GOS89**	7	PB011927	Thermostable organic solvent tolerant lipase *Bacillus sp*. Q5U780 (EC 3.1.1.3)

Reviewed amino acid sequences with the maximal annotation score of 5 and bacterial origin were downloaded from UniProt [27]. Based on matching HMMs from Pfam (Release 27.0) we determined the 4 protein families with the highest homology to the well-characterized proteins from UniProt

**Fig. 3:**
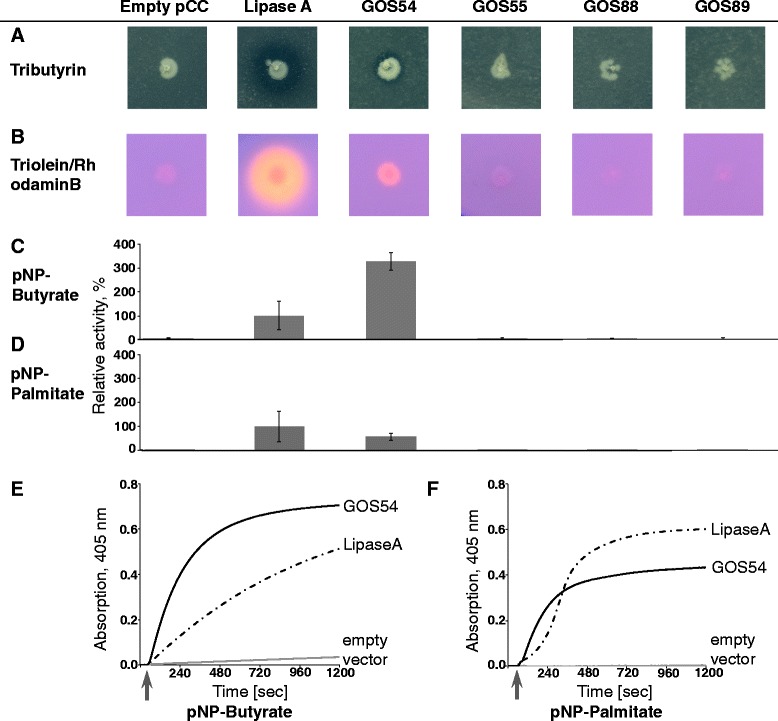
Screening of selected representatives for lipolytic activity. **a** Strains containing empty vector (empty pCC) serving as negative control and expressing Lipase A from *Bacillus subtilis* serving as positive control, as well as representatives for families FUMEFAM011958 (GOS54), FUMEFAM010194 (GOS55), FUMEFAM018084 (GOS88), and FUMEFAM012527 (GOS89) were cultivated on LB agar plates containing 1% tributyrin. Clear halos around the colonies indicate lipolytic activity. **b** The same strains on agar plates containing 1% triolein and 0.001% rhodamin B. Colonies show orange fluorescence under UV light in the presence of lipolytic activity. All plates were incubated for 2 days at 37 °C. **c**-**d** Lipolytic activity of crude extracts from these strains. 4 biological replicates were tested. Crude extract of *E. coli* expressing lipase A from *Bacillus subtilis* was set to 100% activity. **c** Crude extract of strains expressing GOS54 was three times more active against pNP-butyrate than lipase A. **d** In contrast, GOS54 was less active against pNP-palmitate as substrate. **e** Substrate conversion over time was measured continuously over 20 min at 405 nm. *E. coli* with empty pCC vector served as a negative control (*grey line*). Heterologously expressed lipase A from *B. subtilis* served as positive control (*dashed line*). Substrate was added as indicated by an *arrow*. GOS54 hydrolyzes both pNP-butyrate and pNP-palmitate, but prefers the shorter chain-length substrate

We synthesized codon-optimized genes for the representatives of these 4 families and cloned them into an IPTG-inducible *Escherichia coli* expression vector. We then transformed these plasmids into *E. coli* and screened for lipolytic activity using plate-based activity assays. As a positive control, we decided to use LipA, a well-characterized lipase from *Bacillus subtilis*. One of these proteins, GOS54 showed activity on a tributyrin plate, producing a clear halo around the clone, similar to the LipA positive control, indicating the ability of this enzyme to degrade triglycerides containing short-chained fatty acids (Fig. 3a). A triolein-based plate assay showed also activity, albeit to a lesser extent (Fig. 3b). We could verify this activity and GOS54’s preference for short-chained fatty acid esters in an activity assay using p-NP esters of butyrate and palmitate as substrates (Fig. 3c - f). This indicated to us that GOS54 is indeed a lipolytic enzyme and that we can use our representative approach to determine the function of protein classes in the GOS dataset.

## Discussion

**Fig. 4:**
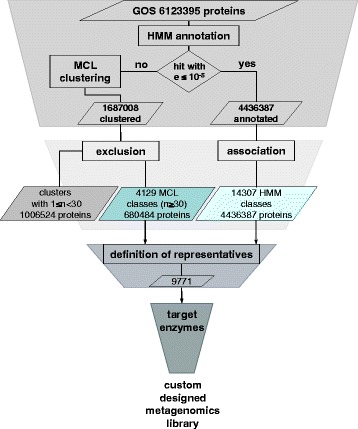
Flow diagram of the classification of proteins of the GOS dataset into families and representatives. First, all protein sequences were annotated using HMM-profiles obtained from PFAM and TIGRFAMs. Proteins that did not match HMMs with scores below the selected threshold were clustered de novo using MCL. Resulting MCL-based classes of small size were excluded. For each HMM-based and MCL-based family that contained sufficient complete sequences, a representative was defined. In this way 9,771 representatives standing in for 4,969,723 proteins were assigned. This set of representatives can then be used to create a custom expression library, which can be screened for a desired target activity

The functional annotation of proteins from metagenomic datasets is challenging. One approach is the distribution of proteins into families based on homology to already known protein families. Using this approach, truly new protein families cannot be discovered. The discovery of novel families unique to the tested dataset can be achieved by a de novo definition of protein families. A de novo definition is typically based on homology of proteins in the data set and computationally substantially slower than an approach based on known HMMs. We therefore decided to use a hybrid approach, assigning families in the GOS metagenomic dataset based on HMMs of known protein families, where possible (see Fig. 4 for a schematic overview). From the remaining proteins that did not contain any known HMMs, we defined protein families using a markov clustering approach [9]. Categorization of the GOS dataset based on HMMs and using the Cd-hit algorithm [19] has been successfully performed before [1], but the resulting data is currently unavailable. The use of the more precise MCL algorithm allowed us to create HMMs based on a substantial number of our de novo-defined families.

Based on the assumption that high homology correlates with an identical function, it should be possible to test just one protein of a family to deduce the function of all members of this family. To define a protein that represents a family in the best way possible, we devised an algorithm that determines the protein with the closest phylogenetic relationship to all other proteins in the family. This determination is based on a phylogenetic tree in which we determine the phylogenetic distance between all members and select the member with the shortest sum of distances to all other members. A similar approach has been used in the COMBREX Project [20]. In this way, we could reduce 4,969,723 proteins that were assigned to families to 9,771 representatives. 1,153,672 proteins are not represented by a representative, either because they were not assigned to a family or because their family contained only incomplete protein sequences. The families, their members and representatives are summarized in Additional file 10.

As a proof of concept of the representative approach we then tested representatives of four families for lipase activity. We selected these representatives based on their homology to well-characterized lipases, because these lipid-degrading enzymes are one of the most commonly used biocatalysts [21]. One representative showed esterase/lipase activity, attesting the usefulness of the representatives approach.

## Conclusion

Here we present a workflow to categorize large metagenomic datasets into protein families. Proteins homologous to known protein families are categorized based on publicly available HMMs. The residual proteins, which do not show homology to known protein families could be categorized de novo. We devised a new algorithm to select one representative from each protein family, which can then be functionally tested in the wet lab. Using representatives from lipolytic families we could verify our approach, discovering the novel esterase GOS54.

## Methods

### GOS dataset

All 6,123,395 hypothetical protein sequences from the GOS dataset were obtained from the NCBI BioProject 13694. Corresponding scaffolds were downloaded from GeneBank (ftp.ncbi.nih.gov/genbank/wgs/gbcon[33–108].seq). This information and the associated annotations were stored in a PostgreSQL database (version 9.0.5) on a Mac mini server (8 × 2 GHz Intel Core i7, 8 GB 1333 MHz DDR3, running Mac OS X Server Lion 10.7.5, Apple, Cupertino, CA). Based on the information contained in the scaffolds, proteins derived from DNA sequences with an unambiguous start and stop codon were defined as “complete”. All other sequences and sequences for which no scaffold information was obtainable were considered “incomplete”.

### HMM profile-based annotation

The GOS data set was annotated using predefined HMMs from PFAM and TIGRFAMs by HMMER 3.0 [8]. The reporting threshold was set to 10^−5^, the output retrieved in table form, all other parameters remained at the default settings.

The 34,833 HMMs from Pfam A and B (Release 27.0) and the 4,424 HMMs from TIGRFAMs (Release 14.0) were obtained from ftp://ftp.ebi.ac.uk/pub/databases/Pfam/, and ftp://ftp.jcvi.org/pub/data/TIGRFAMs/, respectively.

### Family assignment based on HMM annotation

It is possible that individual sequences from the GOS dataset could match multiple HMMs above the reporting threshold. However, we wanted each sequence to be assigned to one and only one protein family. We thus compared the sets of sequences that matched any given HMM with all the other sets of sequences matching the other HMMs by calculating their Jaccard similarity coefficient (Jaccard index). If two sets of sequences matching different HMMs reached an Jaccard similarity coefficient ≥ 0.75, they were combined into one classifier. Multiple HMM-profiles were combined transitively. After all classifiers were defined in this way, each individual sequence was assigned to the classifier, which contained the HMM that it matched with the lowest (i.e. best) threshold. The set of proteins matching a given classifier was defined as protein family and assigned a FUMEFAM number. The alignment of all members of HMM-based families with > 30 members was then optimized using MaxAlign [11] with the command perl -w maxalign.pl -d -f = [$PATH]$I $I.

### All-vs-all BLASTP

To reduce computing time for the blastp program, sequence redundancies were first removed using Cd-hit [19] with the following parameters: cd-hit -i lacking_hmm_seq_14.fasta -o lacking_gos90_14 -c 0.9 -n 5 -M 0 -T 0.

NCBI BLAST 2.2.28+ version was used for a calculation of E-values needed for MCL clustering. To accelerate pairwise comparison, sequences were organized as a BLAST database with a hash index. Protein sequences were split into files with ~10,000 sequences. The BLASTP-based comparison was performed in multi-threads mode on a 32 Core AMD Opteron 6274, 2.2 GHz machine, containing 128 Gb DIMM DDR3 RAM, and 800 Gb hard disk space, running Ubuntu (GNU/Linux 3.13.0–95-generic × 86_64). The e-value was set to 10^−5^, output was set to “table” and the -parse_deflines and -show_GIs parameters were set to “true”.

### Markov clustering

Markov clustering of proteins was performed using the MCL program (version 12–068) according to the protocol by Van Dongen and coworkers [9]. To find an optimized value for the Inflation parameter, 648,901 GOS sequences from 2,356 (~15%) randomly selected HMM-based families were clustered by MCL at values between 1.1, and 6.0. The HMM-based families were then compared to the resulting MCL-based clusters by calculating the Jaccard index. The MCL-based cluster that matched with the highest given Jaccard index was considered the corresponding cluster to that HMM-based family. The highest number of classes which had a Jaccard index > 0.5 with their corresponding cluster was obtained using the inflation parameter 1.1 (see Fig. 1d). We thus used the inflation parameter i = 1.1, other parameters set were: -stream-mirror; -stream-neg-log10; -stream-tf‘ceil (200)’. After the Markov clustering, sequences previously removed using the Cd-hit algorithm were added back to the corresponding cluster, in order to account for all sequences found in the GOS dataset.

### Creation of HMMs and protein families based on MCL clustering

MCL generated 145,314 clusters. To define families, small clusters containing less than 30 protein sequences were removed. To assess the quality of the clusters, alignments were created from the remaining clusters using MAFFT with the default parameters [10]. These alignments were improved by removing sequences that create significant gaps using MaxAlign [11] with the command perl -w maxalign.pl -d -f = [$PATH]$I $I. Clusters, which still contained more than 80% of gaps after that procedure were removed. The number of gaps was calculated using alistat from the biosquid package biosquid_1.9 g + cvs20050121-2_i386 [8]. Clusters that contained less than 10% of complete sequences were also removed. To create a seed alignment for HMM creation, the alignment of the cluster members was further optimized with MAFFT [10] using mafft --reorder --bl 62 --op 2.73 --maxiterate 1000 --globalpair. Poorly aligned regions were removed by Gblocks 0.91b, using Gblocks -t = p -b1 = [half of the numbers of sequences in the alignment] -b2 = [half of the numbers of sequences in the alignment] -b3 = 256 -b4 = 2 -b5 = a -e = .sto. If Gblocks did not identify conserved blocks, the clusters were dismissed. Based on these improved seed alignments, Hidden Markov Models were created using the hmmbuild utility from HMMER 3.0: hmmbuild --fragthresh 1.0 -n [NAME] –o [NAME].out -O [NAME].alig [NAME].hmm [NAME].stockholm. The new HMMs were validated on a test set. In this test set all members from the cluster were added to 80,506 mammalian and plant proteins from RefSeq (see Additional file 7). The HMMs were validated against this test set with

hmmsearch --incE 0.001 -E 0.00001 --tblout [].out [NAME].hmm [NAME]_test_set.fasta.

We then calculated the F1-score for each HMM using the formula

$$ {F}_1=\frac{2\cdot TP}{\left(2\cdot TP+ FP+ FN\right)} $$

where

TP (true positive) = number of family members that matched the HMM;

FP (false positive) = number of RefSeq-based mammalian and plant proteins that matched the HMM. Given that proteins in the GOS dataset are of prokaryotic origin, we argued that matched mammalian and plant proteins would give us an upper limit of false positives;

FN (false negative) = number of family members which did not match the HMM.

Clusters with an F1-score > 0.5 were considered *bona fide* protein families and were assigned a FUMEFAM number.

### Definition of representative family members

In a given family, we defined the relationship distance between each protein pair (i, j) as the sum of the length of all branches directly connecting the pair in a phylogenetic tree. To this end we used the Newick-formated guide tree generated from MAFFT output (mafft --retree 2 --reorder --6merpair –averagelinkage --treeout) based on the members of the final optimized (where applicable) alignment of the family. This guide tree is build using a modified UPGMA algorithm. We then calculated for each protein in the family the sum of all relationship distances to all other proteins using the formula:

$$ Sum\  of\  d istances\ (j) = {\displaystyle \sum_{i=0}^n} d\left( i, j\right) $$

where

d (i,j): distance between sequence i and sequence j in the phylogenetic tree

n: number of sequences in family

The complete protein with the smallest sum of relationship distances was determined as a possible representative for any given family. This process was repeated 100 times using each time 90% of randomly selected sequences from the family. The protein that was selected as possible representative most often was designated as the representative for the family.

### Selection of lipolytic representatives

We selected from the UniProt database bacterial enzymes from the carboxylic ester hydrolases family EC 3.1.1.- with the highest annotation score 5. Enzymes from pathogenic organisms or with oligosaccharides as substrates were excluded manually. The selected proteins were then compared to the PFAM HMMs. The representatives of the families from our database that were classified by the best matching HMMs were retranslated into DNA sequences optimized for *E. coli* codon usage using JCat [22]. These DNA sequences (Table 1) were then synthesized by GeneArt (Thermo Fisher Scientific GENEART GmbH, Regensburg, Germany).

### Protein expression in *Escherichia coli*

Genes were cloned into an IPTG inducible expression vector based on pTAC-MAT-Tag-2 (Sigma– Aldrich, St. Louis, MO, USA) termed pCC and transformed into *Escherichia coli* XL1-Blue using a heat-shock transformation protocol [23]. Strains were grown routinely in LB-medium [24] containing 200 μg/ml ampicillin. For strains used in this study see Table 2.

**Table 2:** Strains used in this study

*Escherichia coli* strain	Genotype	Source
XL1-Blue	recA1 endA1 gyrA96 thi-1 hsdR17 supE44 relA1 lac [F’ proAB laclq ZΔM15 Tn10 (Tetr)]	Stratagene, La Jolla, CA
AK02	XL1-1Blue pCC	This work
AK50	XL1-Blue pCC_*lipA* (pCC containing *lipA* from *B. subtillis* between NdeI and EcoRI restriction sites)	This work
AK70	XL1-Blue pCC_*gos54*	This work
AK71	XL1-Blue pCC_*gos55*	This work
AK72	XL1-Blue pCC_*gos88*	This work
AK73	XL1-Blue pCC_*gos89*	This work

### Plate-based esterase and lipase assays

For all activity assays, at least three biological replicates were tested. To test for lipase activity, LB plates containing 1% (w/v) triolein, 0.001% (w/v) Rhodamin B (Sigma-Aldrich, St. Louis, USA), 200 μg/ml ampicillin, and 100 μM IPTG were prepared [25]. A formation of orange fluorescent halos around colonies on these plates is an indicator of lipase activity. For an esterase activity screening, LB agar plates containing 1% (w/v) tributyrin, 200 μg/ml ampicillin, and 100 μM IPTG were prepared. Esterase activity can be identified by clear halos around colonies on these plates. Overnight cultures of strains were adjusted to an OD_600_ of 2. 2 μl of these cultures, corresponding to ~ 8 × 10^5^ cells were spotted onto the lipase and esterase screening plates.

### Lipolytic activity assays in crude cell extracts

Cell cultures were grown at 37 °C in LB medium containing 200 μg/ml ampicillin. At an OD_600_ between 0.5 and 0.6 the expression of the plasmid-encoded proteins was induced by addition of 500 μM IPTG to the medium. After 3 h, cells were harvested for 1 min at 13,000 × g. Pellets were resuspended in 800 μl 50 mM Tris/HCl buffer, pH 7.3. Bacterial cells were disrupted by sonication using a Vial Tweeter Instrument (Hielscher, Teltow, Germany) at 80% amplitude and cycle of 0.5 for 5 × 30 s with 30 s breaks on ice. Crude extracts were stored at −20 °C. p-Nitrophenyl (p-NP) ester-based activity assays were prepared as described previously [26]. Briefly, activity of crude extracts towards p-NP butyrate and p-NP palmitate (Sigma-Aldrich) was tested in 50 mM Tris/HCl buffer pH 7.3 at 25 °C. The hydrolysis reaction was started by the addition of p-NP ester to a final concentration of 50 μM. The reaction was observed for 20 min, continuously measuring the absorption at 405 nm in a V-650 UV/Vis spectrophotometer (Jasco, Tokyo, Japan). The change of absorption over time (dA/dt) was calculated using the “Enzymatic Reaction Rate” module of the spectrophotometer’s software (Jasco). The activity of crude extract from *E. coli* AK50 (expressing Lipase A from *Bacillus subtilis*) was set to 100%.

## Additional files

Additional file 1: Distribution of HMM-profiles in 890 families created from more than one HMM. (PDF 12 kb)

Additional file 2: Families created based on HMMs and their associated HMMs. (ZIP 226 kb)

Additional file 3: Families created based on HMMs and the corresponding proteins. (ZIP 37.3 mb)

Additional file 4: Classes based on MCL clustering. (ZIP 17128 kb)

Additional file 5: Families based on MCL clustering and the corresponding proteins. (ZIP 6428 kb)

Additional file 6: 4129 novel Hidden Markov Models created in this study. (ZIP 47.9 mb)

Additional file 7: Test set for performance testing of HMMs. (ZIP 6649 kb)

Additional file 8: Performance parameters of the 4129 novel HMMs and their taxonomic specificity. (ZIP 98 kb)

Additional file 9: List of representatives for each family. (ZIP 1506 kb)

Additional file 10: List of all HMM- and MCL-based families and their corresponding proteins. (ZIP 486 mb)

## Abbreviations

DNA: Deoxyribonucleic acid
EMBL: the European molecular biology laboratory
GOS: Global Ocean Sampling
HMM: Hidden Markov Model
MAFFT: Multiple Alignment using Fast Fourier Transform
MCL: Markov Cluster
NCBI: National Center for Biotechnology Information, USA
Pfam: Protein families database
TIGRFAMs: The Institute for Genomic Research protein families database
