# Supplementary material for: In silico approach to designing rational metagenomic libraries for functional studies
Source: BMC Bioinformatics. 2017 May 22;18:267. doi: 10.1186/s12859-017-1668-y (PMC5441078; doi:10.1186/s12859-017-1668-y)
Supplement: Supplementary file 1 — Distribution of HMM-profiles in 890 families created from more than one HMM. (PDF 12 kb) [file 12859_2017_1668_MOESM1_ESM.pdf]

Table S1: Distribution of HMM-based families that combined multiple HMMs

Number of HMMs combined in family	Number of families
2	588
3-5	229
6-10	56
11-20	16
>20	1
